# New Symmetrical U- and Wavy-Shaped Supramolecular H-Bonded Systems; Geometrical and Mesomorphic Approaches

**DOI:** 10.3390/molecules25061420

**Published:** 2020-03-20

**Authors:** Laila A. Al-Mutabagani, Latifah Abdullah Alshabanah, Hoda A. Ahmed, Mohamed Hagar, Khulood A. Abu Al-Ola

**Affiliations:** 1 College of Science, Chemistry Department, Riyadh, Princess Nourah bint Abdulrahman University, Riyadh 11671, Saudi Arabia; Laalmutbagani@pnu.edu.sa (L.A.A.-M); Laalsabanah@pnu.eud.sa (L.A.A.); 2 Faculty of Science, Department of Chemistry, Cairo University, Cairo 12613, Egypt; 3 Chemistry Department, College of Sciences, Yanbu, Taibah University, Yanbu 30799, Saudi Arabia; 4 Faculty of Science, Chemistry Department, Alexandria University, Alexandria 21321, Egypt; 5 Chemistry Department, College of Sciences, Al-Madina Al-Munawarah, Taibah University, Al-Madina 30002, Saudi Arabia; KOla@taibahu.edu.sa

**Keywords:** phenyl nicotinate, supramolecular H-bonding complexes, U-shaped, wavy-shaped, smectic phase, DFT calculations

## Abstract

New mesomorphic symmetrical 2:1 supramolecular H-bonded complexes of seven phenyl rings were prepared between 4-n-alkoxyphenylazobenzoic acids and 4-(2-(pyridin-3-yl)diazenyl)phenyl nicotinate. Mesomorphic studies of the prepared complexes were investigated using differential scanning calorimetry (DSC) and polarizing optical microscopy (POM). Fermi bands of the formed H-bonded interactions were confirmed by FT-IR spectroscopy. Geometrical parameters for all complexes were performed using the density functional theory (DFT) calculations method. Theoretical results revealed that the prepared H-bonded complexes are in non-linear geometry with U-shaped and wavy-shaped geometrical structures; however, the greater linearity of the wavy-shaped compounds could be the reason for their stability with respect to the U-shaped conformer. Moreover, the stable, wavy shape of supramolecular H-bonded complexes (SMHBCs) has been used to illustrate mesomeric behavior in terms of the molecular interaction. The experimental mesomorphic investigations revealed that all complexes possess enantiotropic smectic C phase. Phases were confirmed by miscibility with a standard smectic C (SmC) compound. A comparison was constructed to investigate the effect of incorporating azophenyl moiety into the mesomeric behavior of the corresponding five-membered complexes. It was found that the addition of the extra phenylazo group to the acid moiety has a great increment of the mesophase stability (**T_C_**) values with respect to the monotropic SmC phase of the five aromatic systems to the high stable enantiotropic SmC mesophase.

## 1. Introduction

Geometrical conformations of the liquid crystalline (LC) materials depend on their molecular structures [1,2,3,4,5], which could impact mesophase stability. Hydrogen-bonded (H-bonded) liquid crystalline complexes were reported for the first time by Bradfield, Gray, and Jones [6,7,8]. Thermotropic liquid crystals based on intermolecular hydrogen-bonded interactions are mostly used in display devices as well as sensor applications [9,10,11,12,13]. Most binary H-bonded mixtures are based on pyridine and carboxylic derivatives [14,15,16,17,18,19,20,21,22,23,24]. Recently, our research group [25,26,27,28] started a new research line of LCs by illustrating the behavior of the supramolecular H-bonded (SMHB) complexes of carboxylic acids and pyridine derivatives in the mesophase. These studies focused on the relation between the computational calculations that were estimated from molecular structure predictions and the experimental data evaluated from SMHB mesomorphic behavior. A slight change in the molecular shape of organic materials may change their optical properties and offer new transitions. Moreover, the importance that arises from the possible orientation of heteroatoms in pyridines is to modify existing functions and introduce new desired geometrical characteristics to the organic base molecule. Geometry improvement could be achieved through the introduction of one or two heteroatoms into their architectures. It is known that the heteroatom effect is responsible for changes in the molecular characteristics so that it responds to many demands relevant to optics [29,30,31], material science [32], and electronics [33], as well as the mesomorphic properties of the LCs materials. The ability of 4,4’-Dipyridine to form novel networks through H-bonded complexes has a potential for wide study [34,35,36,37,38]. Further, various changes of spacers [39,40] introduce a conformational flexibility that affects the mesophase type and stability. Previous studies [40] on the thermal and photo-switching characteristics of mesomorphic complexes formed via hydrogen bonding between 4,4’-azabipyridine and 4-alkyloxybenzoic acids were conducted. The presence of dinitrogen atoms with a different position and orientation leads to the redistribution of electron density and changes in the possible aromatic character [41], which allows for various geometrical parameters, such as optoelectronic phenomena.

Mesogenic cores, flexible chains, and terminal groups play an important role in the formation, thermal stability, type, and mesomorphic range of LC compounds. The molecules tend to be oriented in a parallel arrangement as the length of the terminal substituent increases [42]. In addition, the role of the terminal chains influences the heliconical and twist-bend nematic phases [43,44]. Moreover, H-bonded interactions play a significant role in the association of molecules. Recently, density functional theory (DFT) has become an effective tool due to its excellent performance and consistent results with respect to experimental evidence. Furthermore, the molecular geometries and thermal parameters, such as the dipole moment, polarizability, and energy difference between the molecular orbitals of the compound are interesting investigations [5,45,46,47,48,49,50,51,52,53,54]. 

The goal of our present work is to design new structural seven-ring 2:1 supramolecular H-bonded complexes, as well as to conduct a theoretical (DFT) and experimental investigation on the effect of the possible geometrical orientations of prepared binary mixtures. Moreover, our goal is to analyze their mesomorphic, optical, and photophysical properties and correlate the evaluated variables with the predicted geometrical parameters. Finally, we study the effect of incorporating an additional phenylazo group into the previously estimated data of a five-ring system. 
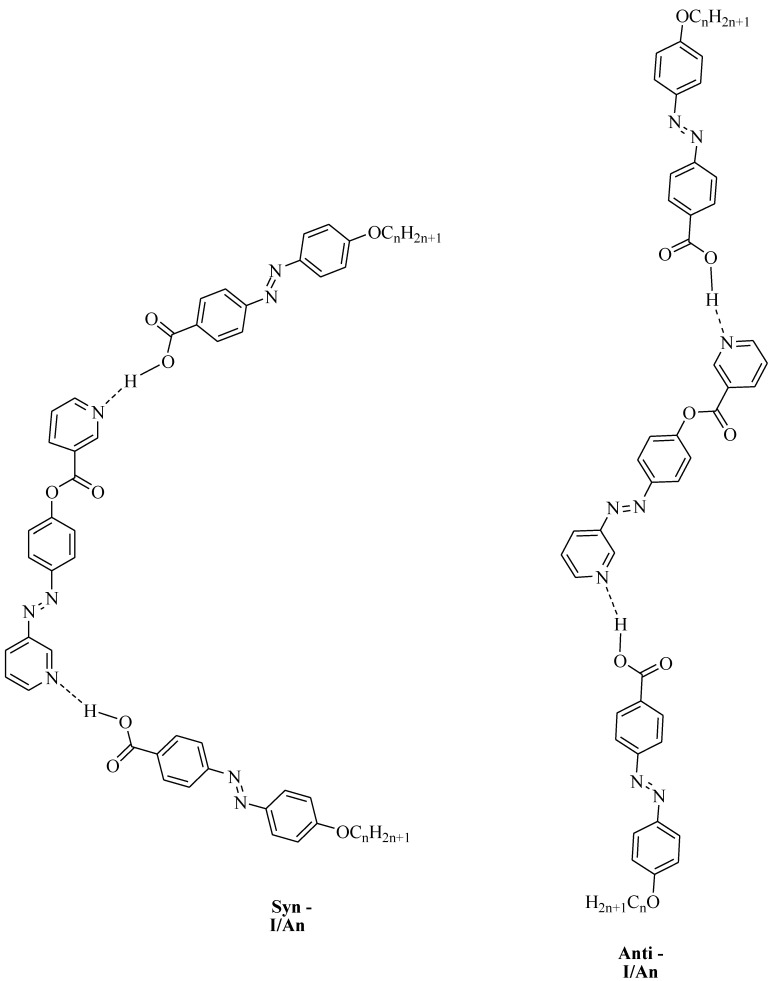


## 2. Experimental Section

### Preparation of 2:1 Symmetrical Supramolecular Complexes

**Scheme 1:**
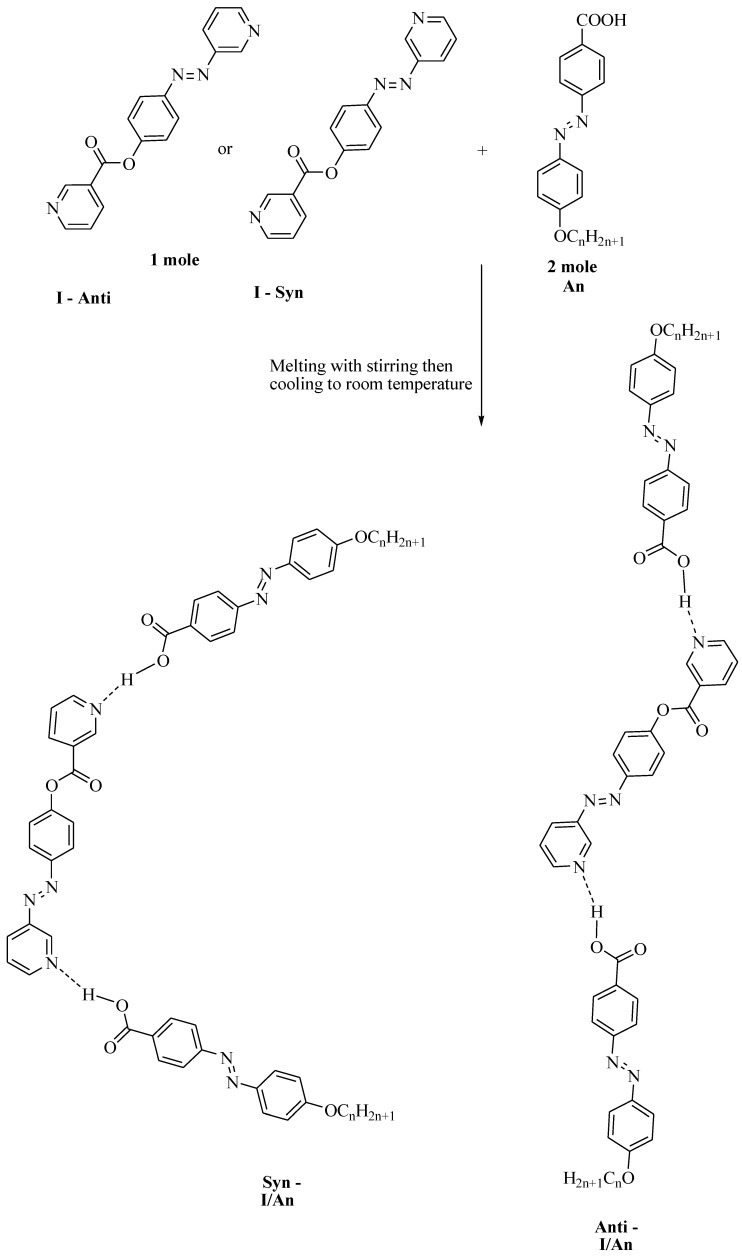
Preparation of 2:1 symmetric supramolecular H-bonded complexes **I/An.**

Symmetrical supramolecular complexes **I/An** were prepared from 1:2 molar ratios of dipyridine **I** and 4-n-alkoxyphenylazobenzoic acids **An**, respectively. The solid mixture was melted by stirring to form an intimate blend and then allowed to cool to room temperature (Scheme 1). 

## 3. Results and Discussion

### 3.1. Theoretical DFT Calculations 

#### 3.1.1. Molecular Geometry

**Figure 1:**
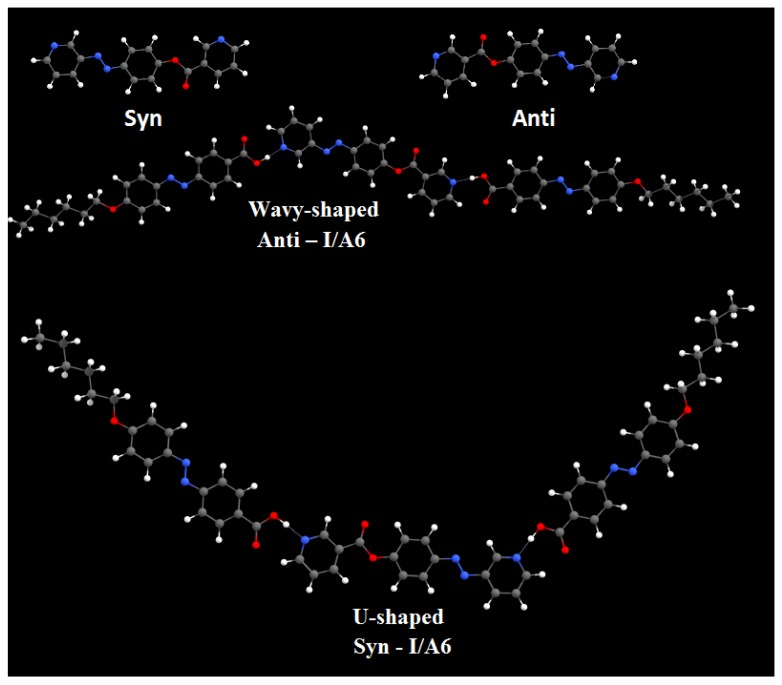
The calculated molecular geometry of Syn-I, Anti-I, Syn-I/A6 and Anti-I/A6.

DFT calculations were carried out using the DFT/B3LYP method at basis set 6-311G for the base and all H-bonded supramolecular complexes I/An. The dinitrogen base was considered to have two conformers, syn and anti-one, and consequently, all H-bonded compounds could exist in both conformers. Figure 1 shows the proposed geometrical structures of both conformers of the H-bonded complexes of the base I with the azo acid A6 of six carbon atoms in the alkoxy chain. 

As shown in Figure 1, the orientation of the nitrogen atom of the symmetrical dinitrogen base has a high impact on the molecular structure of the base as well as the H-bonded complexes. The dinitrogen base I is present in two possible conformers, syn and anti. Even though the base I as well as the acid An are completely linear, the molecular geometry of the H-bonded complexes are non-linear for both conformers. The formed, supramolecular H-bonded complexes, I/An, derived from the syn-isomer of the base, exhibit a non-linear geometry with a U-shaped geometrical structure; however, their corresponding conformational isomeric anti-isomers are also non-linear in geometry with a wavy-shaped structure. 

#### 3.1.2. Thermal Parameters

All thermal parameters were calculated with the same method of calculations under the same set for both conformers of the base I as well as H-bonded complexes (I/An); the data are summarized in Table 1. The syn-isomer of the base is more stable than that of anti-one, with ΔH = 0.2788 kcal mol^−1^. The small value of enthalpy change between the isomers is evidence of the equilibrium existence of both isomers. This higher stability of the syn-isomer could be explained in terms of the better conjugation of the three rings. However, the syn-isomer H-bonded complex I/An is less stable than that of anti-one. The enthalpy difference between the same chain length of the acid (n = 8) is only 0.7222 kcal mol^−1^. The higher stability of anti-isomers of the H-bonded complexes could be illustrated in terms of a greater planarity of the anti-isomer with respect to syn-one. 

#### 3.1.3. Frontier Molecular Orbitals Dipole Moment and Polarizability

**Figure 2:**
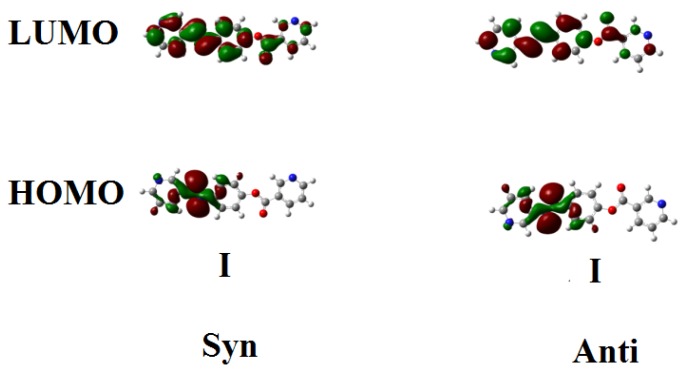
The estimated plots for frontier molecular orbitals of both conformers of dinitrogen base **I**.

**Figure 3:**
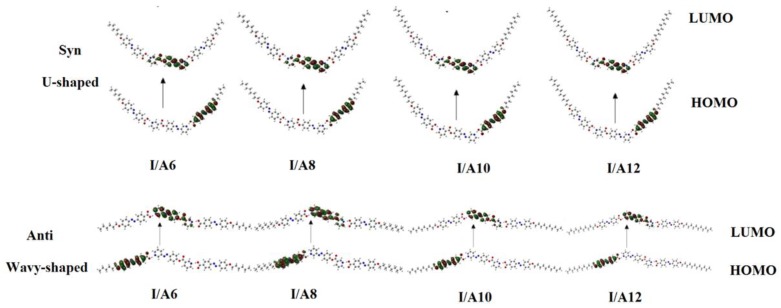
The estimated plots for frontier molecular orbitals of both conformers of the H-bonded complexes **I/An.**

Figure 2 and Figure 3 show the estimated plots for frontier molecular orbitals HOMO (highest occupied) and LUMO (lowest unoccupied) of conformers of the base I, as well as the H-bonded complexes and I/An. The figures emphasize that the electron densities are mainly localized on the 4-n-alkoxyphenylazobenzoic acids for all HOMOs while they shifted to the base in the case of LUMOs. The magnitude of energy difference between the Frontier molecular orbitals (FOMs) could be used in the prediction of the capability of the electron to transfer from HOMOs to LUMOs during any electronic excitation process. The global softness (S) = 1/ΔE is the parameter that predicts the polarizability as well as the sensitivity of the compounds for the photoelectric effects. The higher global softness of the compounds enhances their photoelectric sensitive as well as their polarizability. As shown in Table 2 and Figure 2, the orientation of the nitrogen atoms of the dinitrogen base has little effect on the FMOs’ energy gap as well as on the polarizability and the dipole moment of the H-bonded complexes. The more stable anti-conformer of the H-bonded complex derived from base I is softer than that of the other conformer. Moreover, the lower energy difference of the anti-isomer increases its polarizability. Additionally, the length of the alkoxy chains of the acid component highly affects the polarizability. It has been reported that the more polarizable the compound is, the better the characteristics of the liquid crystalline are for certain applications [55,56,57]. Another important parameter that affects the type of the mesophase and its stability is the dipole moment. It is obvious from Table 2 that the dipole moment of the U-shaped H-bonded complexes is higher than that of the wavy shape derived from the anti-isomer of the dinitrogen base I. 

#### 3.1.4. Molecular Electrostatic Potential

**Figure 4:**
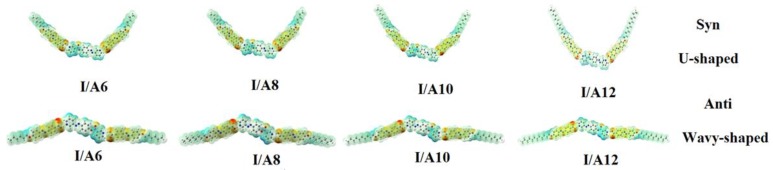
Molecular electrostatic potentials (MEP) of both conformers of the H-bonded complexes **I/An**.

The charge distribution map for both conformers of the H-bonded complexes I/An was calculated with the same method for the same basis sets according to molecular electrostatic potential (MEP) (Figure 4). The negatively charged atomic sites (the red region) were estimated to be localized on carboxylate moiety of the alkoxy azo acid, while the moiety of the dinitrogen base as well as the alkyl chain were predicted to show the least negatively charged atomic sites (blue regions). As shown in Figure 4, the orientation of the charges was affected by the orientation of the nitrogen atoms of the base I, and consequently, this could affect the variation of the mesophase with the orientation of the dinitrogen bases.

### 3.2. Experimental Results 

#### 3.2.1. FT-IR Conformation of Prepared SMHBLCs 

**Figure 5:**
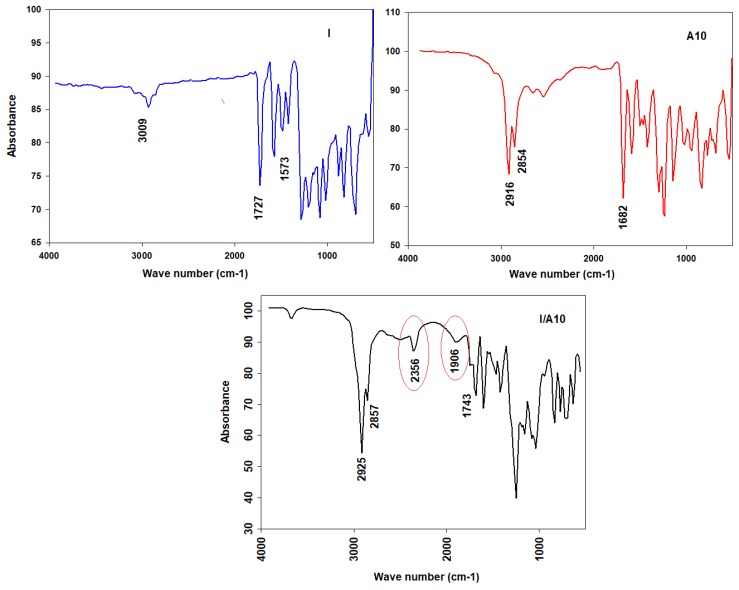
FT-IR spectra of **I**, **A10**, and **I/A10**.

The experimental vibrational bands were examined using the FT-IR absorption spectrum of the dinitrogen base and its H-bonded complex I/A10 (Figure 5). 

For C-H vibrations, the C-H vibrational stretching bands for aromatic rings and aliphatic moiety are located at 3100–2800 cm^−1^. For the C=O of the free alkoxy azobenzoic acid, their vibrational bands are situated at 1680 cm^−1^, and it has been reported that the length of the alkoxy chain has no significant effect of on the vibrational frequency of the C=O group’s stretching vibration. The signal at 1680 cm^−1^ was the stretching vibration of the C=O group of the dimeric form of the 4-alkoxyphenyl azo benzoic acid. The dihydrogen bond between the nitrogen of the dipyridine (I) and the 4-alkoxyphenyl azo benzoic acid (An) of the supramolecular complexes I/An replaces the bis H-bonds of the dimeric form of the acid. For the C=O group of the dipyridine, the formation of the complex has an intensive effect on the stretching vibration of the C=O group of the ester linkage of the dipyridine part; its wave number increases by 16 cm^−1^ from 1727 to 1743 cm^−1^. For Fermi resonance vibration bands, it has been reported [22,58,59,60,61,62,63] that they are an important evidence on the H-bond formation. There are three Fermi bands of the H-bonded OH groups, A-, B-, and C-types. The vibrational band assigned to the A-type Fermi band of complex I/A10 is located under the C-H vibrational peaks at 2935 to 2856 cm^−1^. However, the band at wave numbers lying in the span of 2356 cm^−1^ could be identified as the in-plane bending vibration of the OH group as well as its fundamental stretch (B-type). The stretching bands that are observed at 1906 cm^−1^ were assigned to C-type Fermi bands due to the interaction between the fundamental stretching vibrations of the OH and the overtone of the torsional effect.

#### 3.2.2. Mesomorphic and Optical Investigations 

**Figure 6:**
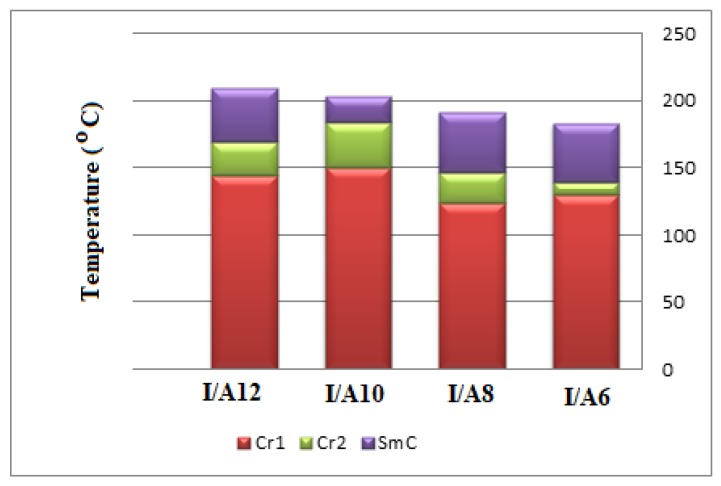
Graphical DSC transitions of 2:1 SMHB complexes, **I/An**.

**Figure 7:**
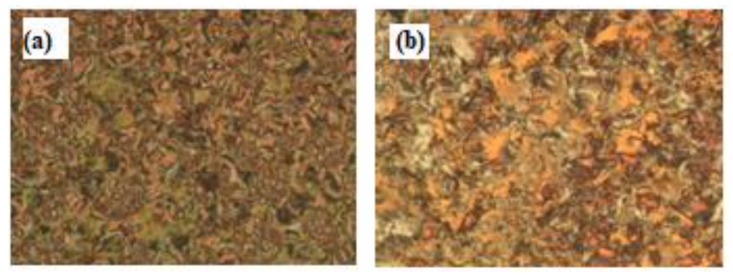
Polarizing optical microscopy (POM) smectic C (SmC) phase texture upon heating for 2:1 supramolecular complex: (**a**) **I/A8** at 185 ^o^C and (**b**) **I/A12** at 197 ^o^C.

Interestingly, we must study the mesomorphic and optical behavior of the prepared supramolecular H-bonded complexes, which have seven aromatic rings (I/An). Investigations of complexes were carried by DSC, and textures under polarizing optical microscopy (POM) confirmed the data evaluated from DSC. Results of the transition temperatures that associate enthalpy and normalized entropy of mesophase transitions for all the analyzed di-symmetric SMHB complexes I/An, as derived from DSC measurements, are collected in Table 3. Mesomorphic transition temperatures are graphically represented in Figure 6 in order to study the effect of the terminal alkoxy chain length of the acid component on the mesophase behavior. During the second heating and cooling scans, all SMHB mixtures (I/An) showed an enantiotropic smectic C phase with a good range of thermal stabilities. The POM investigations confirmed the smectic C (SmC) mesophase textures (Figure 7). The data values of Table 1 and Figure 4 reveal that enantiotropic smectic C mesophase was only observed for all investigated 2:1 mixtures, and their thermal stabilities increased by increasing the terminal alkoxy acid chain length. Moreover, irregular trends were observed in the melting point transitions of the complexes. Further, all investigated complexes exhibit two solid crystalline phases (given as Cr1 and Cr2), followed by an SmC phase. In order to confirm that the SmC phase is the only mesophase exhibited by all complexes, a binary phase diagram was constructed between I/A6 and the smectogenic 4-hexadecyloxy benzoic acid as an example. The diagram is depicted in Figure S4 as supplementary data; the phase diagram exhibits only the enantiotropic SmC phase.

It was reported [64] that the dipyridine derivative I is non-monomorphic and converted directly from a crystalline solid state to isotropic liquid states at 150.7 °C without displaying any liquid crystal phase. Meanwhile, the pure alkoxyphenylazobenzoic acids An exhibited an smectic C mesophase, followed by a narrow range of nematic phase (N) [28]. However, all prepared 2:1 supramolecular complexes I/An showed only the SmC phase with relatively higher ranges of mesophase stability for the complex I/A8 (~44.5 °C) and the lower value for I/A10 (~19.5 °C). In addition, the SmC transition stability decreases with the increment of the acid chains (n), and thus, the terminal length and the mesogenic core of the H-donor component have an important role in enhancing the stability of the formed phase. Moreover, it was found that the polarity difference between H-donors and H-acceptor affects the hydrogen bonding strength and increment of the molecular anisotropy, and it promotes broadening of the mesophase range [28]. However, the polarity of both components of the mixture is not affected by the length of the terminal alkoxy chain. From a theoretical calculations study, it can be concluded that the higher dipole moment increases the lateral interaction with respect to the terminal one, and this could be another good explanation of the smectic mesophase formation for the H-bonded complexes I/An. Furthermore, the wavy shape of the anti-isomer of the H-bonded complexes I/An explains the smectic C mesophase. The lateral interaction of the wavy shape could be higher than the terminal interaction; consequently, the slightly ordered smectic phase is a predominating phase. As the terminal chain length increases, the lateral interaction will be enhanced, and the stability of the SmC phase increases. This geometry permits the maximum terminal alkoxy chain aggregations to show the smectogenic mesophase. Moreover, the increment of the chain length highly enhanced the estimated stability of the complexes, whereas the predicted energy deceases by lengthening the chains; however, the difference in the energy between the proposed complexes is the same.

**Figure 8:**
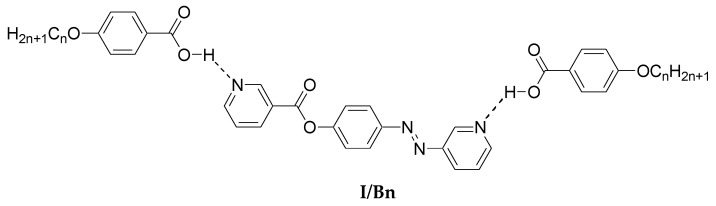

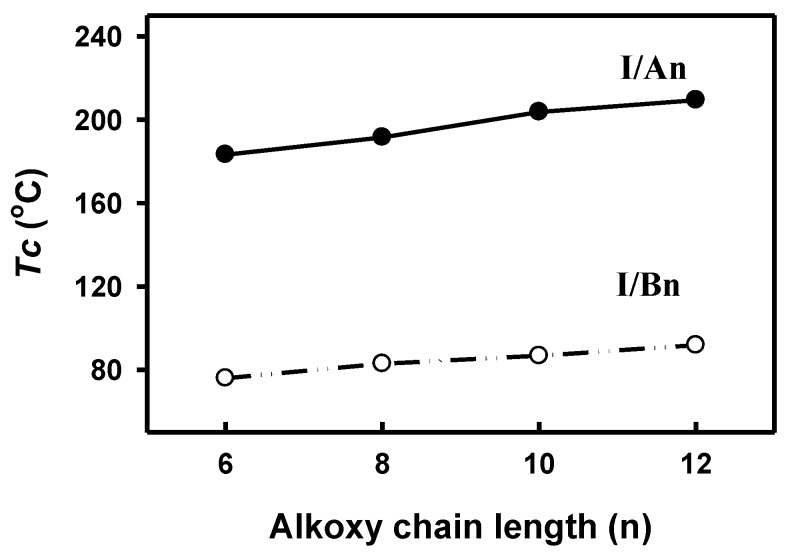
Comparison between present SMHB complexes **I/An** (●) and five-ring complexes **I/Bn** (○) in thermal mesophase stability.

In order to investigate the effect of the addition of an extra phenylazo group moiety into the previously investigated complexes, I/Bn [64], on the mesophase thermal stability (T_C_), a comparison was made with the data reported before [64] and is illustrated here in Figure 8. As can be seen from Figure 8, the addition of the extra phenylazo group into the acid component resulted in a great increase of the Tc values from the monotropic SmC phase of I/Bn to the enantiotropic high stable SmC mesophase for I/An. These increases are attributed to the increase of polarizability of the whole molecule, as well as the increase of rigidity and the aspect ratio, which in turn lead to the increase of intermolecular interactions between molecules.

#### 3.2.3. Entropy Changes

Normalized entropy of smectic C transitions (*∆S/R*), driven from DSC, were calculated for the present symmetrical supramolecular H-bonded complexes I/An; the results are collected in Table 3. The results indicate that a random trend of the entropy changes with the terminal alkoxy chain lengths. The variation in the entropy change with acid terminals may be attributed to a change of molecular interactions between molecules, which are affected by the dipole moment, polarizability, rigidity, aspect ratio (length/breadth ratio), and geometrical shape of molecules. These factors may contribute to the conformational, orientational, and translational entropies of the molecule in different amounts. Moreover, the relationship between the change in the entropy, *∆S/R*, and the type of the mesophase could be explained in terms of the wavy shape of the H-bonded complexes I/An, as previously discussed from the DFT calculations. Moreover, it could be emphasized from DFT that the increment of the alkoxy chain length increased the predicted total electronic stability and resulted in enhancing the smectic mesophase stability effect of the prepared H-bonded complex I/An. These results could be explained in terms of the enhancement of the alkoxy chain wing aggregation as well as the higher degree of parallel interactions. The longer the terminal lengthens, the more conformational orientations of the molecules, the higher the total thermodynamic energy and the smectic C stability (T_C_) are. Finally, incrementing the alkoxy chain length of I/An does not impact either the orientation or the amount of the charges in MEP calculations, and consequently, it could be used to illustrate the smectic C phase observed for all H-bonded complexes with little increment of the mesophase stability of a longer chain length.

## 4. Conclusions

Here, we reported the symmetrical 2:1 supramolecular H-bonded complexes of the seven phenyl ring system. Mesomorphic and optical investigations were carried out by DSC and POM. H-bonded interactions were confirmed via the formation of Fermi bands using FT-IR spectroscopy. DFT theoretical calculations were performed to estimate the thermal and geometrical parameters for the present SMHB complexes and their individual components. Results revealed that the geometrical structures of the present complexes are non-linear and have U-shaped and wavy-shaped structures. Moreover, the wavy-shaped structure is more stable than the U-shaped conformer. The mesomorphic study revealed that the symmetric orientation of dinitrogen atoms in the base moiety has an important role in the observation of the enantiotropic SmC mesophase with a broad range of stability than the previously reported five-ring complexes system. Furthermore, the wavy shape of the SMHB complexes has been used to illustrate the mesomeric behavior in terms of the molecular interaction.

## Figures and Tables

**Table 1:** Thermal parameters (Hartree/Particle) of both conformers of the base I as well as the H-bonded complexes I/An.

Conformer	Syn	Anti	Syn				Anti			
Parameter	I	I	I/A6	I/A8	I/A10	I/A12	I/A6	I/A8	I/A10	I/A12
**E_corr_**	0.262	0.262	1.0	1.1	1.3	1.4	1.0	1.1	1.3	1.4
**ZPVE**	−1024.112	−1024.112	−3168.2	−3325.3	−3482.4	−3639.6	3168.2	−3325.3	−3482.4	−3639.6
**E*_tot_***	−1024.094	−1024.093	−3168.1	−3325.2	−3482.4	−3639.5	−3168.1	−3325.2	−3482.4	−3639.5
**H**	−1024.093	−1024.092	−3168.1	−3325.2	−3482.4	−3639.5	−3168.1	−3325.2	−3482.4	−3639.5
**G**	−1024.163	−1024.163	−3168.3	−3325.4	−3482.6	−3639.7	−3168.3	−3325.4	−3482.6	−3639.7

**Table 2:** FMO energies (a.u.), polarizability, α (Bohr3), and dipole moment μ (Debye) of both conformers of the supramolecular H-bonded (SMHB) complexes I/An.

Parameter	Syn				Anti			
	I/A6	I/A8	I/A10	I/A12	I/A6	I/A8	I/A10	I/A12
**E_LUMO_**	−0.12030	−0.12035	−0.12055	−0.12056	−0.12081	−0.12080	−0.12080	−0.12078
**E_HOMO_**	−0.22075	−0.22073	−0.22068	−0.22066	−0.21410	−0.22135	−0.22131	−0.22126
**ΔE_HOMO-LUMO_**	0.10045	0.10038	0.10013	0.1001	0.09329	0.10055	0.10051	0.10048
**Total µ**	2.6897	2.7634	2.7941	2.8109	1.9935	1.4576	2.0176	2.0260
**Polarizability α**	893.01	941.85	988.48	1036.07	902.07	951.13	999.38	1047.24

**Table 3:** Phase transition temperatures (°C), enthalpy of transitions (kJ/mol), and normalized transition entropy of supramolecular H-bonded complexes **I/An**.

Compound	*T*_Cr1-Cr2_	*T*_Cr2-SmC_	*∆H*_Cr2-SmC_	*T*_SmC-I_	*∆H*_SmC-I_	*∆S/R*
**I/A6**	130.4	139.7	38.67	183.2	3.91	1.03
**I/A8**	124.0	147.0	27.21	191.5	2.98	0.77
**I/A10**	150.2	184.2	21.99	203.7	2.70	0.68
**I/A12**	144.7	169.2	28.24	209.4	3.68	0.92

Abbreviations: ***T***_SmC-I_ = Smectic C to isotropic liquid transition. ***∆H***_SmC-I_ = Smectic C to isotropic liquid phase transition. ***∆S/R*** = Normalized entropy transition.

## Supplementary Materials

The following are available online, **Scheme 1.** Synthesis of 4-(2-(pyridin-3-yl)diazenyl)phenyl nicotinate (**I**). *Figure S1: 1H-NMR of 4-(2-(pyridin-3-yl)diazenyl)phenyl nicotinate* (**I**) Figure S2: DSC thermograms upon second heating /cooling cycles for supramolecular complex **I/A10**. Figure S3: DSC thermograms upon second heating /cooling cycles for supramolecular complex **I/A12**. Figure S4: Binary phase diagram of the complex I/A6 with 4-hexadecyloxybenzoic acid; Cr1 to: smectic C transition (○); Cr2 to smectic C transition (●); and SmC to isotropic liquid transition (■).
