# Supplementary material for: New Symmetrical U- and Wavy-Shaped Supramolecular H-Bonded Systems; Geometrical and Mesomorphic Approaches
Source: Molecules. 2020 Mar 20;25(6):1420. doi: 10.3390/molecules25061420 (PMC7144929; doi:10.3390/molecules25061420)
Supplement: Supplementary file 1 [file molecules-25-01420-s001.pdf]

Supplementary Data

New Symmetrical U- and Wavy- shaped Supramolecular H-bonded Systems; Geometrical and Mesomorphic Approaches

Laila A. Al-Mutabagani[1], Latifah Abdullah Alshabanah[1],Hoda A. Ahmed [2, 3]*, Mohamed Hagar [3, 4]*and Khulood A. Abu Al-Ola[5]

1. College of Science, Chemistry Department, Riyadh, Princess Nourahbint Abdulrahman University, Saudi Arabia. (L.A.A.M) Laalmutbagani@pnu.edu.sa, (L.A.A.) Laalsabanah@pnu.eud.sa.
2. Faculty of Science, Department of Chemistry, Cairo University, Cairo, Egypt. (HA) ahoda@sci.cu.edu.eg.
3. Chemistry Department, College of Sciences, Yanbu, Taibah University, Yanbu 30799 Saudi Arabia
4. Faculty of Science, Chemistry Department, Alexandria University, Alexandria, Egypt. (MH) mohamedhaggar@gmail.com
5. Chemistry Department, College of Sciences, Al-Madina Al-Munawarah, Taibah University, Al-Madina 30002,

*Correspondence: M. Hagar, mhagar@taibahu.edu.sa; H.A. Ahmed, ahoda@sci.cu.edu.eg

Material and methods

Nicotinic acid, *N,N′*-dicyclohexylcarbodiimide (DCC), phenol, 3-aminopyridine, 4-dimethylaminopyridine (DMAP) and 3-aminopyridine were purchased from Aldrich (Wisconsin, USA). All solvents used are pure grade and purchased from Aldrich (Wisconsin, USA).

TA Instruments Co. Q20 Differential Scanning Calorimeter (DSC; USA) were using for calorimetric measurements. The DSC was calibrated using the melting temperature and enthalpy of indium and lead. DSC investigation was carried out for small samples (2–3 mg) placed in aluminum pans. All measurements were achieved at a heating rate of 10°C/min in inert atmosphere of nitrogen gas (30 ml/min) and all transition recorded from the second heating scan.

Transition temperatures for the individual components and their 2:1 associated complex, were determined by DSC, and the types of the mesophase identified by a standard polarized optical microscope (POM, Wild, Germany) attached with Mettler FP82HT hot stage. The temperature is measured by thermocouple attached to the temperature controller. Measurements were made twice, and the results have accuracy in transition temperature within ± 0.2°C.

Characterizations

Purity of **I** was checked with thin-layer chromatography using TLC and elemental analyses. The structure was confirmed by FTIR (Nicolet iS 10 Thermo scientific), and ^{1}H-NMR spectroscopy (Varian EM 350L 300 MHz spectrometer, Oxford, UK).

Computational Method and calculations

The theoretical calculations for the investigated compounds were carried out by Gaussian 09 software [1]. DFT/B3LYP methods using 6-31G (d,p) basis set was selected for the calculations. The geometries were optimized by minimizing the energies with respect to all geometrical parameters without imposing any molecular symmetry constraints. The structures of the optimized geometries had been drawn with Gauss View [2]. Moreover, the calculated frequencies were carried out using the same level of theory. The frequency calculations showed that all structures were stationary points in the geometry optimization method with none imaginary frequency.

Preparation of dipyridine-based derivative

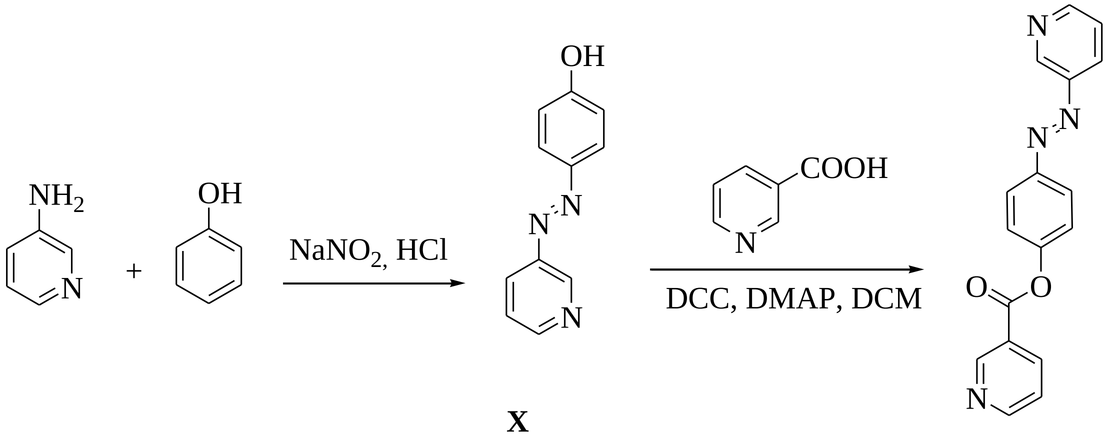

X

I

Scheme 1. Synthesis of4-(2-(pyridin-3-yl)diazenyl)phenyl nicotinate (**I**).

Synthesis of4-(2-(pyridin-3-yl)diazenyl)phenol (**X**)

This was prepared according to the method described by Zhang *et al.* [3] from 3-aminopyridine and phenol. A mixture of (2.5 g, 26.5 mmol) phenol in sodium hydroxide 10% (50 ml) was cooled to 0 °C.Another coldsolution of 3-aminopyridine (2.4 g, 27 mmol) in 6 molar HCl was added drop wise to a cold aqueous solution of sodium nitrite (2.0 g, 30 mmol) in H_2O (20 ml). Thelatersolution, the diazonium solution, was added to the former, sodium phenoxide,with stirring within 10 minutes and keeping the

reaction temperature below 5 ^{o}C. Subsequently, the complete precipitation of the product was performed by neutralization of the reaction mixture with sodium carbonate till pH ≈ 6. The obtained orange dye was purified by recrystallization from hot aqueous ethanol (1:1).The yield of the product was 75%. [4]

***Synthesis of4-(2-(pyridin-3-yl)diazenyl)phenyl nicotinate*(I)**

An equal molar ratios of 4-(2-(pyridin-3-yl)diazenyl)phenol (**X**) (1.99,0.01 mole) and nicotinic acid (1.23 g, 0.01 mole) was dissolved in 25 ml dry methylene chloride. A catalytic amount of 4–dimethylaminopyridine (DMAP) and 0.02 mole of *N*, *N′*–dicyclohexylcarbodiimide (DCC,) was added. The reaction mixture was kept stirring for 72 hours at room temperature. The separated byproduct, *N,N*-dicyclohexylurea, was filtered off. The evaporation of the filtrate afforded the ester derivative, the product was recrystallized twice from ethanol (**Scheme 1**).

Yield: 94.0 %; mp 150.7 ^{o}C, FTIR (ύ, cm^{-1}): 3042 (=CH stretching), 1724 (C=O), 1578 (C=N), 1488 (C–O$_{Asym}$), 1275 (C-O $_{Sym}$). ^{1}H NMR (400 MHz, $CDCl_3$): δ/ppm: ^{1}H NMR (400 MHz, $CDCl_3$): δ/ppm: 9.47 (s, 1H, Py), 9.30 (s, 1H, Py), 8.93 (d, J = 3.8 Hz, 1H, Py), 8.81 (d, J = 3.3 Hz, 1H, Py), 8.60 (d, J = 8.1 Hz, 1H, Py), 8.55 (d, J = 7.9 Hz, 1H, Py), 8.13 (d, J = 8.6 Hz, 2H, Ph), 7.86 (dd, J = 7.7, 5.2 Hz, 1H, Py), 7.58 (dd, J = 7.7, 5.2 Hz, 1H, Py), 7.50 (d, J = 8.7 Hz, 2H, Ph). Elemental analyses: Found (Calc.): C, 67.07 (67.10); H, 3.95 (3.97); N, 18.40 (18.41).

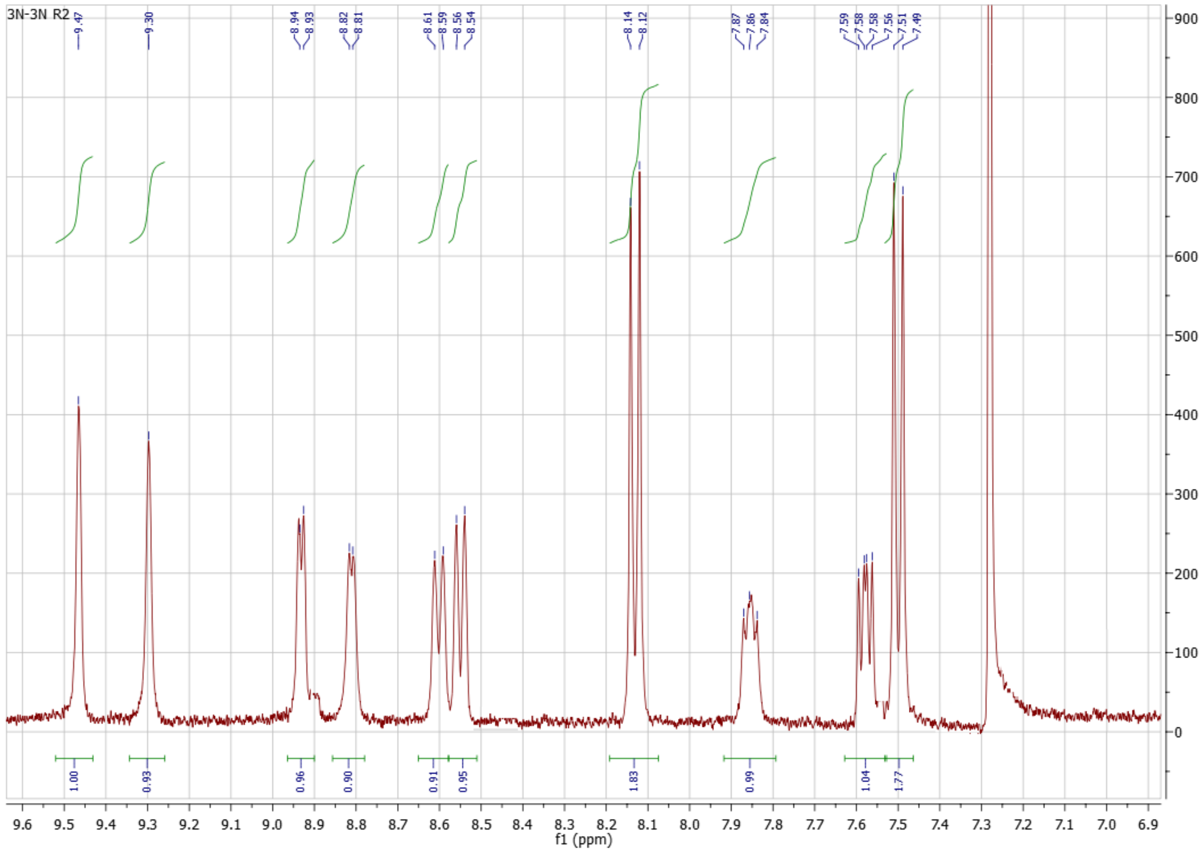

Figure S1: 1H-NMR of 4-(2-(pyridin-3-yl)diazenyl)phenyl nicotinate (**I**)

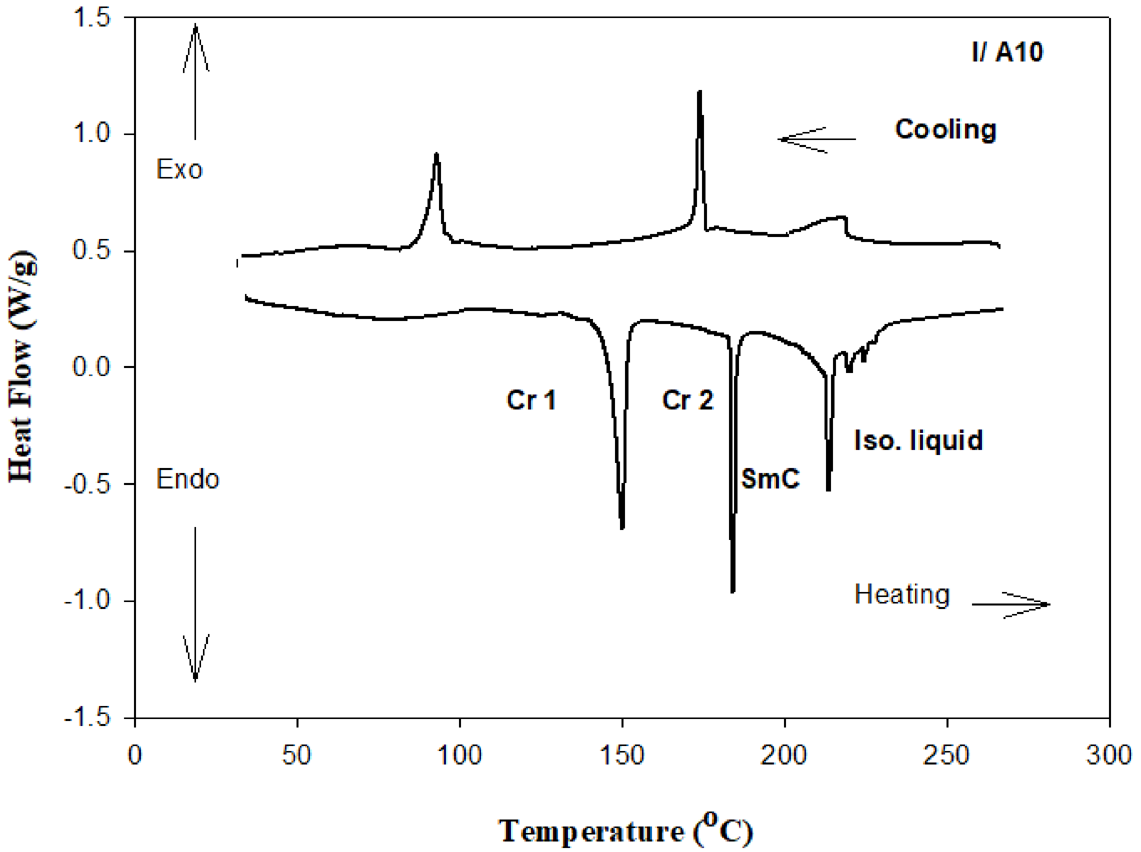

Figure S2: DSC thermograms upon second heating /cooling cycles for supramolecular complex **I/A10**.

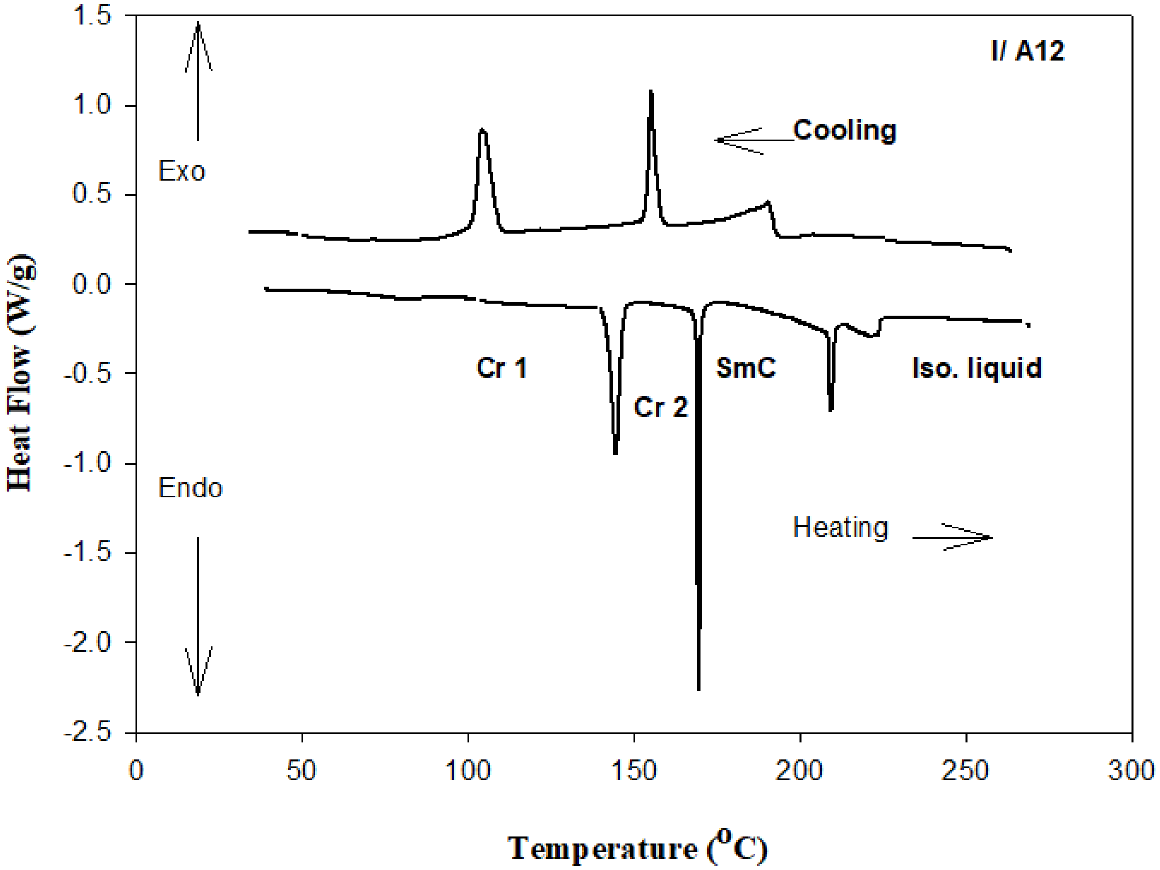

Figure S3: DSC thermograms upon second heating /cooling cycles for supramolecular complex **I/A12**.

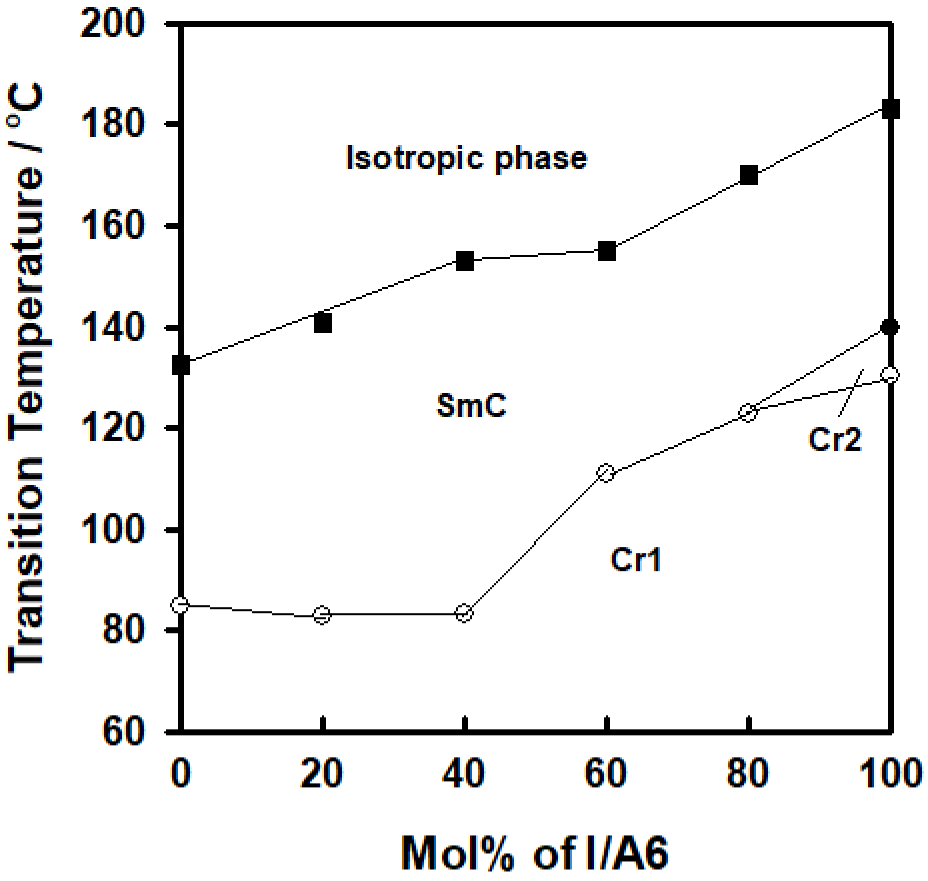

Figure S4: Binary phase diagram of the complex **I/A6**with4-hexadecyloxybenzoic acid; Cr1 to: smectic C transition(○) ; Cr2 to smectic C transition(●); and SmC to isotropic liquid transition (■).

References

1. Frisch, M.; Trucks, G.; Schlegel, H. B.; Scuseria, G.; Robb, M.; Cheeseman, J.; Scalmani, G.; Barone, V.; Mennucci, B.; Petersson, G., Gaussian 09, revision a. 02, gaussian. *Inc., Wallingford, CT* **2009,** 200.
2. Dennington, R.; Keith, T.; Millam, J., GaussView, version 5. **2009**.
3. Song, X.; Li, J.; Zhang, S. Liq. Cryst. 2003, 30, 331–335.

4. Naoum MM, Fahmi AA, Refaie AA, et al. Novel hydrogen-bonded angular supramolecular liquid crystals. Liq Cryst. 2012;39:47–61. doi:10.1080/02678292.2011.611262
